# Vaccines That Reduce Viral Shedding Do Not Prevent Transmission of H1N1 Pandemic 2009 Swine Influenza A Virus Infection to Unvaccinated Pigs

**DOI:** 10.1128/JVI.01787-20

**Published:** 2021-01-28

**Authors:** Helen E. Everett, Pauline M. van Diemen, Mario Aramouni, Andrew Ramsay, Vivien J. Coward, Vincent Pavot, Laetitia Canini, Barbara Holzer, Sophie Morgan, the Dynamics sLoLa Consortium, Mark E. J. Woolhouse, Elma Tchilian, Sharon M. Brookes, Ian H. Brown, Bryan Charleston, Sarah Gilbert

**Affiliations:** a APHA-Weybridge, New Haw, Addlestone, United Kingdom; b The Jenner Institute, Nuffield Department of Medicine, University of Oxford, Oxford, United Kingdom; c Usher Institute, Ashworth Laboratories, University of Edinburgh, Edinburgh, United Kingdom; d The Pirbright Institute, Pirbright, Woking, United Kingdom; St. Jude Children's Research Hospital

**Keywords:** influenza A, pH1N1, pig, vaccine, transmission

## Abstract

Swine influenza A virus (swIAV) infection causes substantial economic loss and disease burden in humans and animals. The 2009 pandemic H1N1 (pH1N1) influenza A virus is now endemic in both populations. In this study, we evaluated the efficacy of different vaccines in reducing nasal shedding in pigs following pH1N1 virus challenge. We also assessed transmission from immunized and challenged pigs to naive, directly in-contact pigs. Pigs were immunized with either adjuvanted, whole inactivated virus (WIV) vaccines or virus-vectored (ChAdOx1 and MVA) vaccines expressing either the homologous or heterologous influenza A virus hemagglutinin (HA) glycoprotein, as well as an influenza virus pseudotype (S-FLU) vaccine expressing heterologous HA. Only two vaccines containing homologous HA, which also induced high hemagglutination inhibitory antibody titers, significantly reduced virus shedding in challenged animals. Nevertheless, virus transmission from challenged to naive, in-contact animals occurred in all groups, although it was delayed in groups of vaccinated animals with reduced virus shedding.

**IMPORTANCE** This study was designed to determine whether vaccination of pigs with conventional WIV or virus-vectored vaccines reduces pH1N1 swine influenza A virus shedding following challenge and can prevent transmission to naive in-contact animals. Even when viral shedding was significantly reduced following challenge, infection was transmissible to susceptible cohoused recipients. This knowledge is important to inform disease surveillance and control strategies and to determine the vaccine coverage required in a population, thereby defining disease moderation or herd protection. WIV or virus-vectored vaccines homologous to the challenge strain significantly reduced virus shedding from directly infected pigs, but vaccination did not completely prevent transmission to cohoused naive pigs.

## Abstract (precis)

This study was designed to determine whether vaccination of pigs with conventional WIV or virus-vectored vaccines reduces pH1N1 swine influenza virus shedding following challenge and can prevent transmission to naive in-contact animals. Even when viral shedding was significantly reduced following challenge, infection was transmissible to susceptible cohoused recipients.

## INTRODUCTION

Influenza virus infection imposes a substantial disease burden on both humans and animals and has a considerable economic impact. In pigs, swine influenza A virus (swIAV) infections contribute to the porcine respiratory disease complex, with consequences for animal welfare and financial loss to the global pig industry. Pigs are also a key intermediate host that can support the comingling of virus strains originating from diverse species. Consequently, circulation of swIAV in pigs has been identified as a source of virus diversification, which may contribute to zoonotic risk (reviewed in references 1 and 2). Enzootic swine influenza viruses show high genetic and antigenic diversity globally, in comparison to human-origin influenza viruses. The worldwide emergence of the 2009 pH1N1 influenza A virus lineage, which is now endemic in both pigs and humans, has contributed substantially to this diversity (reviewed in references 2 and 3). However, there is no systematic global surveillance of swIAV genetic drift (gradual accumulation of mutations) or genetic shift (exchange of genetic segments by the process of reassortment) and the potential associated changes in antigenic properties. Both mechanisms contribute to the wide genetic diversity within pig herds, both regionally within countries and internationally, creating challenges for disease control strategies.

Vaccination of pigs remains the primary method of swine influenza disease control (reviewed in references [Bibr B4][Bibr B5][Bibr B7]). Available swIAV vaccines, including for the pH1N1 strain, are commonly whole inactivated virus (WIV) formulations, but they can show limited efficacy in the field due in part to the complexity of strain choice and lack of regular updates to antigen composition (2, 4). With the limitations of current vaccines and continual virus evolution, control of swIAV remains a challenge. Efforts to improve efficacy of vaccines for animals and humans are focused on inducing broad-spectrum immunity to reduce the clinical impact of disease and ultimately provide protection, as well as preventing transmission (reviewed in references 4 and 8).

We have previously shown that a homologous influenza WIV vaccine formulated in adjuvant significantly reduced virus shedding after pH1N1 virus challenge in pigs, whereas a heterologous WIV vaccine did not (9). We have also investigated the efficacy of aerosol delivery of S-FLU, an influenza pseudotype vaccine candidate limited to a single cycle of replication through inactivation of the hemagglutinin (HA) signal sequence. S-FLU has been found to induce a robust T cell response in the lung, but a minimal antibody response to HA (10, 11). We have shown that S-FLU reduced viral load in nasal swabs and the lung after challenge with a partially matched virus strain in pigs (12). However, after heterosubtypic challenge, S-FLU reduced lung pathology in pigs, but not viral load (13). We have also previously evaluated influenza vaccines constructed using replication-deficient, recombinant viral vectors based on chimpanzee adenovirus Oxford 1 (ChAdOx1) and modified vaccinia virus Ankara (MVA) expressing both the viral nucleoprotein (NP) and matrix protein (M1) (NP+M1), with or without different constructs of HA, in animal models (14–16) and human clinical trials (17). Mice immunized with NP+M1 and group 2 chimeric HA (cHA) molecules in these viral vectors showed improved protection against influenza A virus challenge compared to either antigen (NP+M1 or cHA) alone (16). In addition, priming with ChAdOx1-NP+M1 and boosting with MVA-NP+M1 reduced virus titers in the respiratory tract of ferrets after H3N2 challenge (14). Coadministration of MVA-NP+M1 with HA protein or following a prime then boost with ChAdOx1-NP+M1 also induced T cell responses to NP and M1 in pigs, although no challenge was performed in these studies (15). Furthermore, clinical trials with prime-boost combinations of MVA-NP+M1 and ChAdOx1-NP+M1 in young and old subjects have demonstrated strong vaccine immunogenicity in both groups (17).

To control swIAV in pig herds and reduce the risk of zoonotic events, a vaccine that prevents shedding and transmission of the virus is required. However, in most studies, vaccine efficacy is assessed by measuring immune responses, viral shedding, clinical signs, and lung pathology after live virus challenge, and few have evaluated whether vaccines can prevent onward transmission (18). In the present study, we evaluated the efficacy of WIV, single-cycle S-FLU, and virus-vectored ChAdOx1 and MVA vaccines, matched or mismatched for the HA, against challenge with pH1N1 virus. We also investigated the subsequent direct contact transmission of the challenge virus to naive unimmunized pigs, thereby addressing the impact of vaccination on infection, as well as infectiousness or ability to disseminate infection.

## RESULTS

### Experimental design of immunization, challenge, and contact transmission studies.

**FIG 1:**
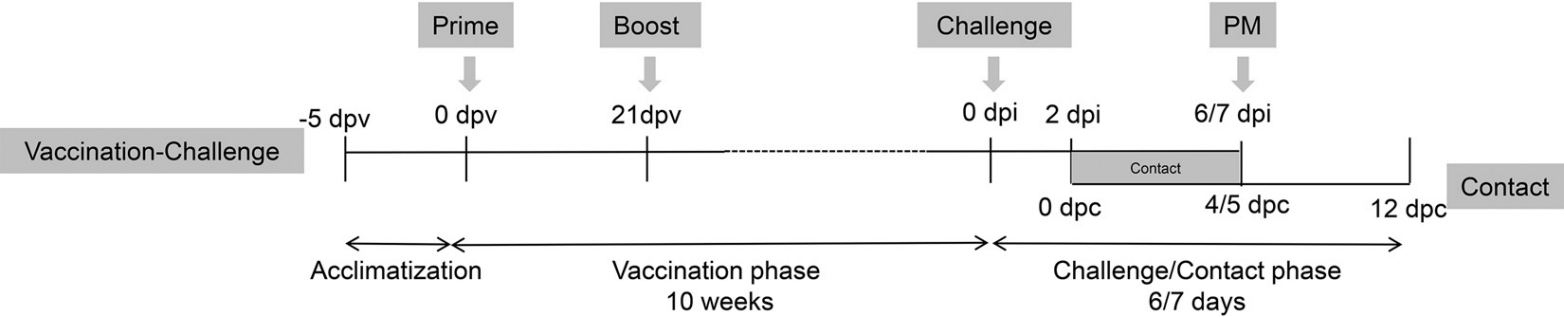
Study design. Groups of five pigs were prime-boost vaccinated on days 0 and 21 postvaccination (dpv) while cohoused during the vaccination phase. For the challenge phase, pigs were housed in separate groups and challenged with the pH1N1 strain A/swine/England/1353/2009 10 weeks after the prime vaccination. Pigs were monitored daily postinoculation (dpi) until postmortem (PM) sampling on 6 or 7 dpi. On 2 dpi, five naive unvaccinated contact pigs were introduced into each group and were monitored daily until 12 days postcontact (dpc) at the end of the study. Inoculated and unvaccinated pigs remained in contact for a total of 4 or 5 days.

Immunization, influenza A virus challenge, and contact transmission studies were carried out as detailed in Fig. 1. Six vaccines were evaluated in two separate studies for logistical reasons (Table 1). Groups of five pigs were immunized twice with a 3-week interval. Study 1 included the following groups: G1, mock vaccine allantoic fluid as vehicle control administered intramuscularly (i.m.) (Cont); G2, homologous WIV pH1N1 vaccine i.m. (WIV_hom_); G3, heterologous WIV H1_av_N1 vaccine i.m. (WIV_het_); and G4, H3N2 influenza A virus pseudotype delivered by aerosol (S-FLU). In study 2, pigs were immunized i.m. by group as follows: G5, ChAdOx1 prime and MVA boost containing an unrelated antigen (Cont_Ad/MVA_); G6, ChAdOx1 prime and MVA boost expressing homologous HA, NP, and M1 (Ad_hom_/MVA_hom_); G7, MVA expressing the homologous HA, NP, and M1 (MVA_hom_); and G8, MVA expressing homologous NP and M1 and heterologous H1_av_-origin HA (MVA_het_). Immunizations did not induce any adverse reactions. One pig in the Cont_Ad/MVA_ group was removed on clinical grounds unrelated to the study.

**TABLE 1:** Characteristics of the vaccines used in this study^*a*^

Vaccination (group)	Antigen origin	Vaccine dose		Adjuvant
		Prime	Boost	
Cont (G1)	None (vehicle only: allantoic fluid and PBS)			TS6
WIV_hom_ (G2)	A/swine/England/1353/2009 pH1N1 (clade 1A.3.3.2) HA	1,024 HAU	1,024 HAU	TS6
WIV_het_ (G3)	A/swine/England/453/2006 H1_av_N1 (clade 1C.2) HA	1,024 HAU	1,024 HAU	TS6
S-FLU (G4)	A/Switzerland/9725293/2013 H3N2 (clade 3C.3a) HA	S-FLU, 1.5 × 10^8^ TCID_50_/ml	S-FLU, 1.5 × 10^8^ TCID_50_/ml	
Cont_Ad/MVA_ (G5)	Ebola virus (Zaire) glycoprotein	ChAdOx1, 5 × 10^8^ IU/ml	MVA, 1.5 × 10^8^ PFU/ml	
Ad_hom_/MVA_hom_ (G6)	A/swine/England/1353/2009 pH1N1 (clade 1A.3.3.2) HA	ChAdOx1, 5 × 10^8^ IU/ml	MVA, 1.5 × 10^8^ PFU/ml	
MVA_hom_ (G7)	A/swine/England/1353/2009 pH1N1 (clade 1A.3.3.2) HA	MVA, 1.5 × 10^8^ PFU/ml	MVA, 1.5 × 10^8^ PFU/ml	
MVA_het_ (G8)	A/swine/England/453/2006 H1_av_N1 (clade 1C.2) HA	MVA, 1.5 × 10^8^ PFU/ml	MVA, 1.5 × 10^8^ PFU/ml	

a The vaccines used in this study were whole inactivated virus (WIV), influenza pseudotype (S-FLU), or virus-vectored constructs based on ChAdOx1 (Ad) or modified vaccinia virus Ankara (MVA). Cont, control; WIV_hom_, homologous WIV; WIV_het_, heterologous WIV; Cont_Ad/MVA_, control with unrelated antigen; Ad_hom_/MVA_hom_, homologous Ad/MVA with pH1N1 HA+NP+M1; MVA_hom_, homologous MVA with pH1N1 HA+NP+M1; MVA_het_, heterologous MVA with HA_av_+pH1N1 NP+M1.

Ten weeks after the first immunization (7 weeks after the boost), pigs were challenged intranasally (i.n.) with 1 × 10^7^ 50% tissue culture infective doses (TCID_50_) of pH1N1, monitored daily, and removed for necropsy at 6 or 7 days postinoculation (dpi). Unvaccinated naive “contact” pigs (*n* = 5) were housed from 2 dpi with each vaccinated and challenged group. Contact was maintained for 4 to 5 days, when the directly pH1N1-challenged pigs were removed. The contact pigs were monitored for a total of 12 days postcontact (dpc). Clinical signs were mild or not apparent for the duration of the study in all inoculated and contact groups, and clinical scores transiently reached no more than 2 out of a possible maximum of 20 for several pigs for the duration of the study (data not shown).

### Virus shedding after direct challenge and contact animal introduction.

**FIG 2:**
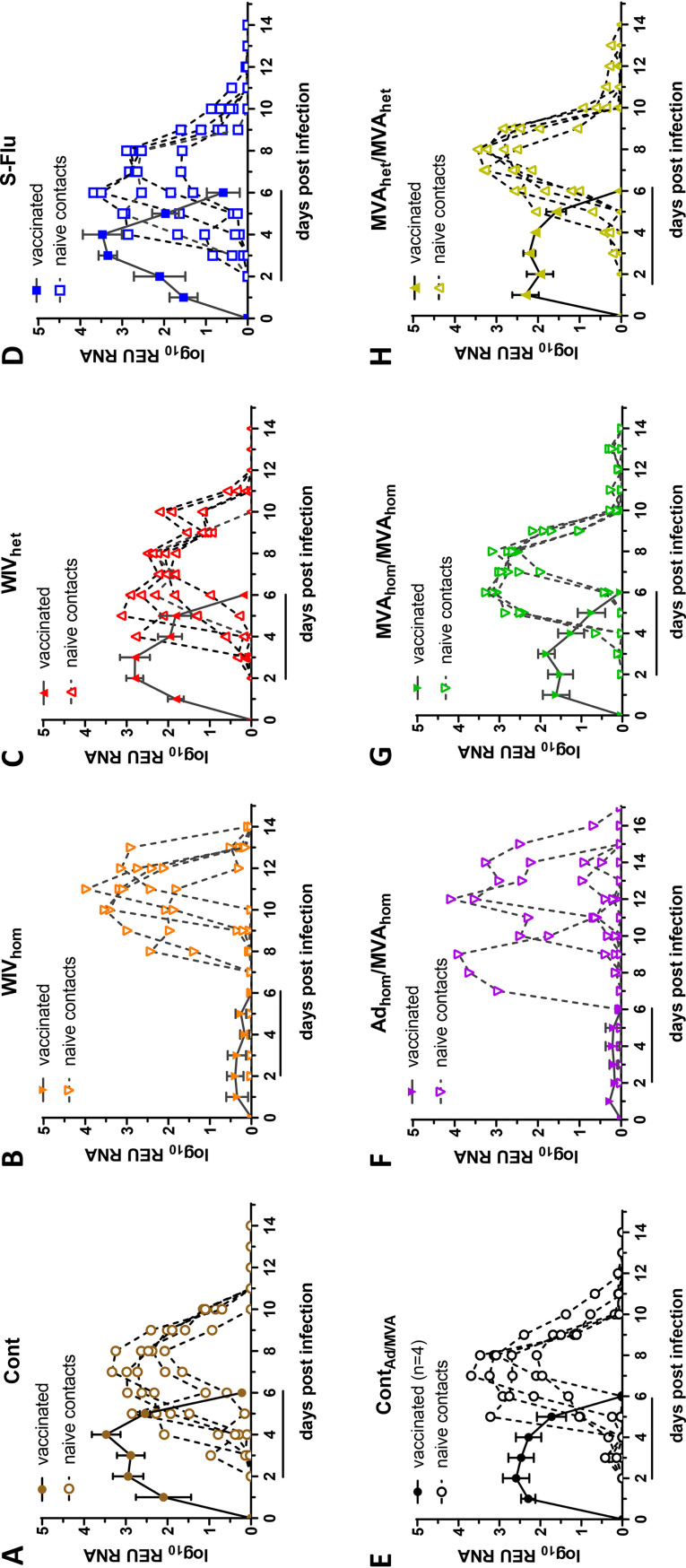
Viral shedding. In each group (A to H), viral RNA shedding following challenge was assessed daily in nasal swabs by RRT-qPCR and is expressed as mean log_10_ relative equivalent units REU. The mean REU (± SEM) is shown for the vaccinated and challenged pigs in each group (solid lines), as well as the individual shedding profiles for the naive pigs in these groups (dotted lines). Horizontal lines under the *x* axis denote the contact period.

Nasal shedding of viral RNA was monitored daily in the immunized, directly virus-challenged and the contact pigs by quantitative real-time reverse transcription-PCR (RRT-qPCR) (Fig. 2A to H). All directly pH1N1-inoculated pigs shed virus from 1 dpi, except for the WIV_hom_- or Ad_hom_/MVA_hom_-immunized groups. These groups given the homologous vaccine to the challenge virus showed minimal virus shedding at the lower limit of quantification (Fig. 2B and F). Mean viral shedding in the directly infected control-immunized animals (Cont) in study 1 (Fig. 2A) or the vector control Cont_Ad/MVA_-immunized group in study 2 peaked between 2 and 5 dpi (Fig. 2E). Significant differences (*P* < 0.05) were observed between these groups on days 4 and 5 dpi, but as these studies were done on different occasions and there was no unimmunized control, it is not known whether this difference was due to a study effect or possible nonspecific adjuvant effect of the viral vector.

Two days after pigs were challenged, each group was cohoused with naive, unimmunized pigs, and nasal swabs were obtained daily to monitor virus shedding. Transmission occurred in all groups, including the WIV_hom_- and Ad_hom_/MVA_hom_-immunized groups, despite significant reduction of virus shedding after direct pH1N1 challenge. However, in the WIV_hom_ group, two contact animals started shedding virus at 6 dpc, and the onset of shedding was later in the remaining animals. In the Ad_hom_/MVA_hom_ group, one contact animal started shedding at 5 dpc and the remainder between 8 and 10 dpc. In contrast, in all other groups, the majority of contacts started shedding 2 to 3 dpc. It is possible that in the WIV_hom_ and Ad_hom_/MVA_hom_ groups, the contact animals showing initial shedding infected the other naive pigs in their group. However, once infected, most contact pigs shed virus to the same level despite the delay, except for two contact animals in the Ad_hom_/MVA_hom_ group.

**FIG 3:**
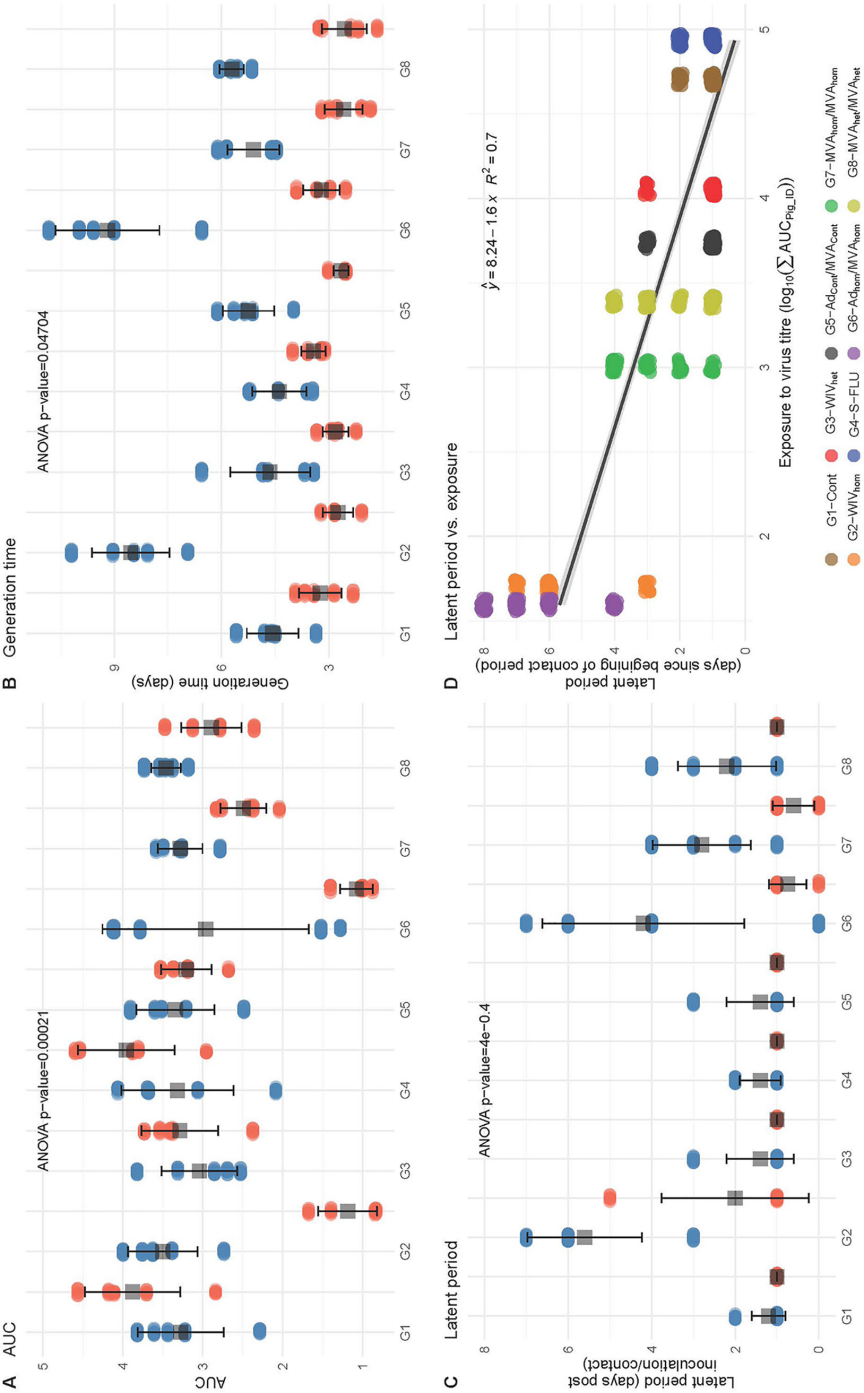
Statistical modeling. Nasal shedding of viral RNA (A to C) from vaccinated, inoculated pigs (red) and naive contact pigs (blue) was modeled for all groups as follows: G1, Cont; G2, WIV_hom_; G3, WIV_het_; G4, S-FLU; G5, Cont_Ad/MVA_; G6, Ad_hom_/MVA_hom_; G7, MVA_hom_; and G8, MVA_het_. For each parameter, circles represent the individual value, the gray squares show the mean, and the black error bars indicate the mean ± SD. Analysis is shown for (A) the area under the curve (AUC), which provides a measure of total viral RNA shedding, (B) the generation time (*T_g_*), which is the time interval between the onset of virus shedding in a primary case and in its secondary case, and (C) the latent period, which is the interval between inoculation or contact and first detection of nasal shedding of viral RNA. The relationship between the latent period and the exposure to virus (D) is shown for each treatment group, with virus exposure computed as the sum of the AUC in log scale for the vaccinated pigs in a given treatment group.

Statistical analysis of the nasal shedding profile of viral RNA supported these conclusions. There were clear pairwise differences (summarized in Table 2) between the vaccinated and control groups in terms of *V*_max_ (the peak viral titer shed), *T*_max_ (the time to reach *V*_max_ following inoculation or contact), the area under the curve (AUC) as a measure of total amount of virus shed, and *T_g_* (the generation time, or time interval between the onset of virus shedding in directly inoculated pigs in relation to naive contact pigs). The *P* values are shown for vaccinated pigs (Table 3) and contact pigs (Table 4). The variance between the different groups (Fig. 3) was also assessed. AUC values indicative of total viral RNA shedding (Fig. 3A and Table 3) as well as *V*_max_ measuring peak viral RNA shedding (Table 3) were significantly different for the WIV_hom_- and Ad_hom_/MVA_hom_-immunized groups compared to the other groups. Correspondingly, the generation time (*T_g_*), representing the mean interval between inoculation of the immunized pigs and detection of shedding in the naive contact pigs, was significantly longer for the pigs in contact with these two WIV_hom_- and Ad_hom_/MVA_hom_-immunized groups (Fig. 3B and Table 4). *T*_max_, or the time from contact to peak virus shedding, was also significantly longer for these two contact pig groups (Table 4). In addition, there was a significant correlation between the lower AUC value in the vaccinated pigs, indicative of reduced viral shedding, and a longer latent period (interval between virus exposure and first detection of virus) in the contact pigs in the WIV_hom_ and Ad_hom_/MVA_hom_ groups (Fig. 3C and D). These results show that only the WIV_hom_ and Ad_hom_/MVA_hom_ vaccines significantly reduced shedding after direct pH1N1 challenge. This did not prevent transmission to unimmunized contact pigs, although the infection of contact animals was delayed.

**TABLE 2:** Summary of statistical analysis as shown by pairwise permutation test significance

Parameter^*a*^	Result for^*b*^:	
	Vaccinated	Contact
Latent period	No significant difference	No significant difference
*T*_end_	No significant difference	No significant difference
*D_v_*	No significant difference	No significant difference
*V*_max_	**Significant difference**	No significant difference
AUC	**Significant difference**	No significant difference
*T*_max_	No significant difference	**Significant difference**
*T_g_*	No significant difference	**Significant difference**

a Definitions of parameters are as follows: Latent period, interval between inoculation or contact and first detection of virus; *T*_end_, time to last detection of virus; *D_v_*, duration of shedding; *V*_max_, peak viral titer shed; AUC, area under the curve; *T*_max_, time to reach *V*_max_ following inoculation or contact; *T_g_*, generation time (i.e., time interval between the onset of virus shedding in a primary case and in its secondary case).

b Significant differences are highlighted in boldface.

**TABLE 3:**
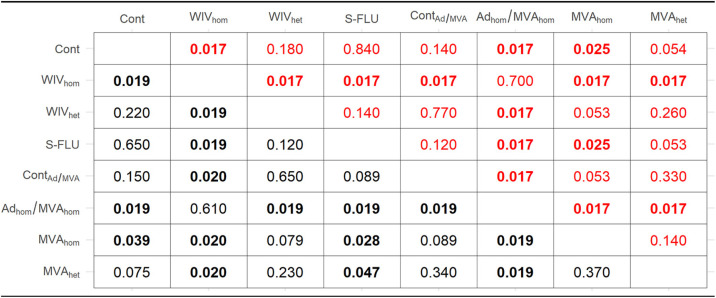
*P* value results of pairwise permutation tests to compare the AUC and the peak viral titer shed in vaccinated pigs^*a*^

a Shown are the *P* value results of pairwise permutation tests to compare the area under the curve (AUC [red]) and the peak viral titer shed (*V*_max_ [black]) in vaccinated pigs. Significant adjusted *P* values of <0.05 are shown in boldface.

**TABLE 4:**
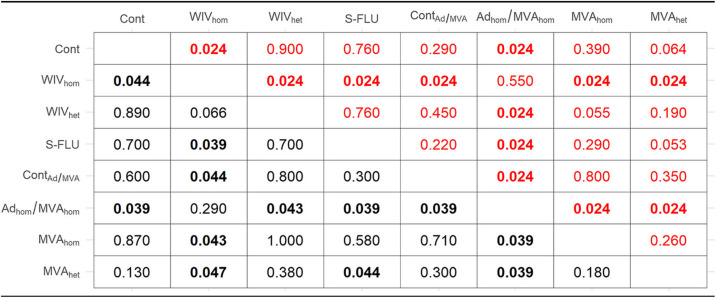
*P* value results of pairwise permutation tests to compare the generation time and the time from inoculation or contact to peak virus shedding in contact pigs^*a*^

a Shown are the *P* value results of pairwise permutation tests to compare the generation time, or the time interval between the onset of virus shedding in a primary case and in its secondary case (*T_g_* [red]), and the time from inoculation or contact to peak virus shedding (*T*_max_ [black]) in contact pigs. Significant adjusted *P* values of <0.05 are shown in boldface.

### Humoral response.

**FIG 4:**
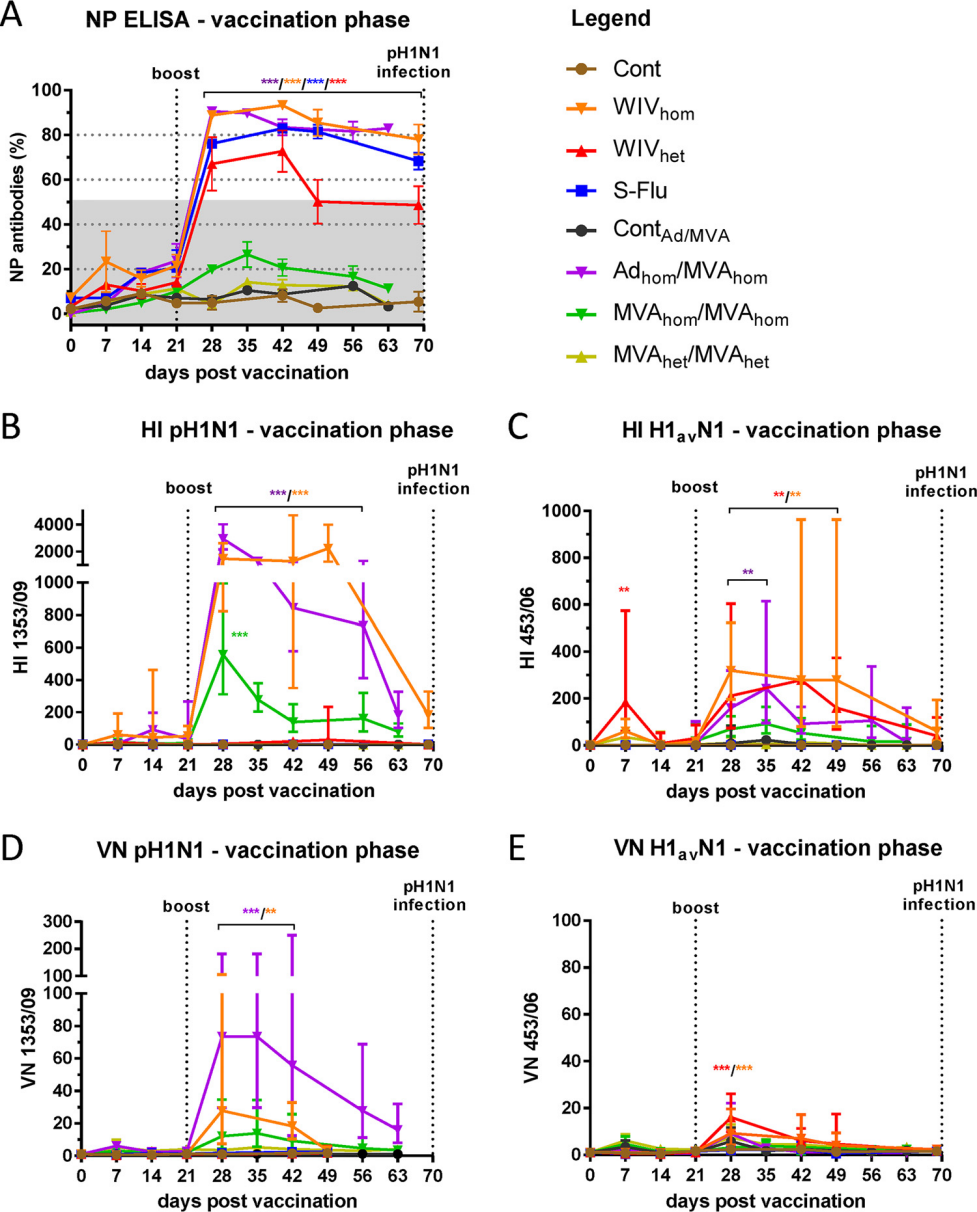
Humoral immune response during the vaccination phase. Longitudinal serum samples were assessed by influenza A virus nucleoprotein (NP) competition ELISA, hemagglutination inhibition (HI), and virus neutralization (VN) assays. (A) Anti-NP antibody response is expressed as the mean (± SEM) of the inverse of the percentage of competition: 1 − (OD_sample_/OD_negative_) × 100%. Results below the 50% threshold (gray shading) are considered negative. Antibody levels in the WIV_hom_-, WIV_het_-, S-Flu-, and Ad_hom_/MVA_hom_-immunized groups above the 50% threshold are significantly higher (*P* < 0.0001) than those in the control groups (Cont and Cont_Ad/MVA_). HI and VN titers against (B and D) the homologous antigen, pH1N1 (A/swine/England/1353/2009), and (C and E) the heterologous antigen, H1_av_N1 (A/swine/England/453/2006), are shown as the geometric mean ratios (GMRs ± geometric SD) for the titers for each pig relative to the corresponding 0-dpv sample. **, *P* < 0.001, and ***, *P* < 0.0001, by the color corresponding to the group, as indicated under “Legend.”

Prior to immunization, influenza virus-specific antibodies were not detected using both NP enzyme-linked immunosorbent assay (ELISA) and hemagglutination inhibition (HI) assays (Fig. 4). A robust and significant (*P* < 0.0001) anti-influenza NP immune response, indicative of replicating virus, was detected following boost immunization in the WIV_hom_, WIV_het_, S-FLU, and Ad_hom_/MVA_hom_ groups (Fig. 4A). The antibody responses in the MVA_hom_ and MVA_het_ groups were below the threshold of the assay. As expected, no antibody response to NP was detected in the vehicle control (Cont) and vector control (Cont_Ad/MVA_) pigs before infection. Antibody responses to immunization were also measured by HI assay using the homologous (clade 1A.3.3.2) and heterologous (1C.2) HA vaccine antigens (Fig. 4B and C). Pigs immunized with WIV_hom_ or Ad_hom_/MVA_hom_ mounted significant (*P* < 0.0001) antibody responses to the homologous HA antigen after boost immunization compared to the vehicle and vector control groups (Cont and Cont_Ad/MVA_), with average geometric mean ratio (GMR) titers of 2,941 and 1,470, respectively, peaking at 28 days postvaccination (dpv) (Fig. 4B). MVA_hom_ immunization elicited a lower HI antibody titer, with a significant peak GMR of 557 at 28 dpv (*P* < 0.0001). HI antibody titers in pigs immunized with the WIV_het_ measured against the cognate (1C.2) HA antigen showed a significant (*P* = 0.008) response a week after prime immunization and peaked at a GMR of just under 300 a week after boost (*P* = 0.002). The WIV_hom_- and Ad_hom_/MVA_hom_-vaccinated pigs elicited comparable titers of cross-reactive antibodies after boost (*P* < 0.005) (Fig. 4C). Pigs in the Cont_Ad/MVA_ and S-FLU groups did not produce influenza A virus-specific antibodies, as expected. Surprisingly, MVA_het_-vaccinated animals did not produce antibodies to either antigen detectable by HI before virus infection. Virus neutralization (VN) antibody titers elicited by immunization were considerably lower than the anti-NP ELISA and HI antibody levels. Antibodies elicited by homologous vaccination in the WIV_hom_ and Ad_hom_/MVA_hom_ groups on 28 to 42 dpv (*P* < 0.001 or *P* < 0.0001 for the respective vaccine groups) and to a lesser extent MVA_hom_ (not significant), neutralized the pH1N1 challenge virus strain (geometric mean titers of 28, 74, and 14, respectively) (Fig. 4D). Neutralizing antibodies against the heterologous HA antigen were elicited by the cognate WIV_het_ vaccine and reached a peak GMR titer of 16 at 28 dpv (*P* < 0.0001). Cross-reactive antibodies were also detected in the WIV_hom_ group (*P* < 0.0001) (Fig. 4E) but could not be detected in the other groups above the limit of sensitivity of the assay.

**FIG 5:**
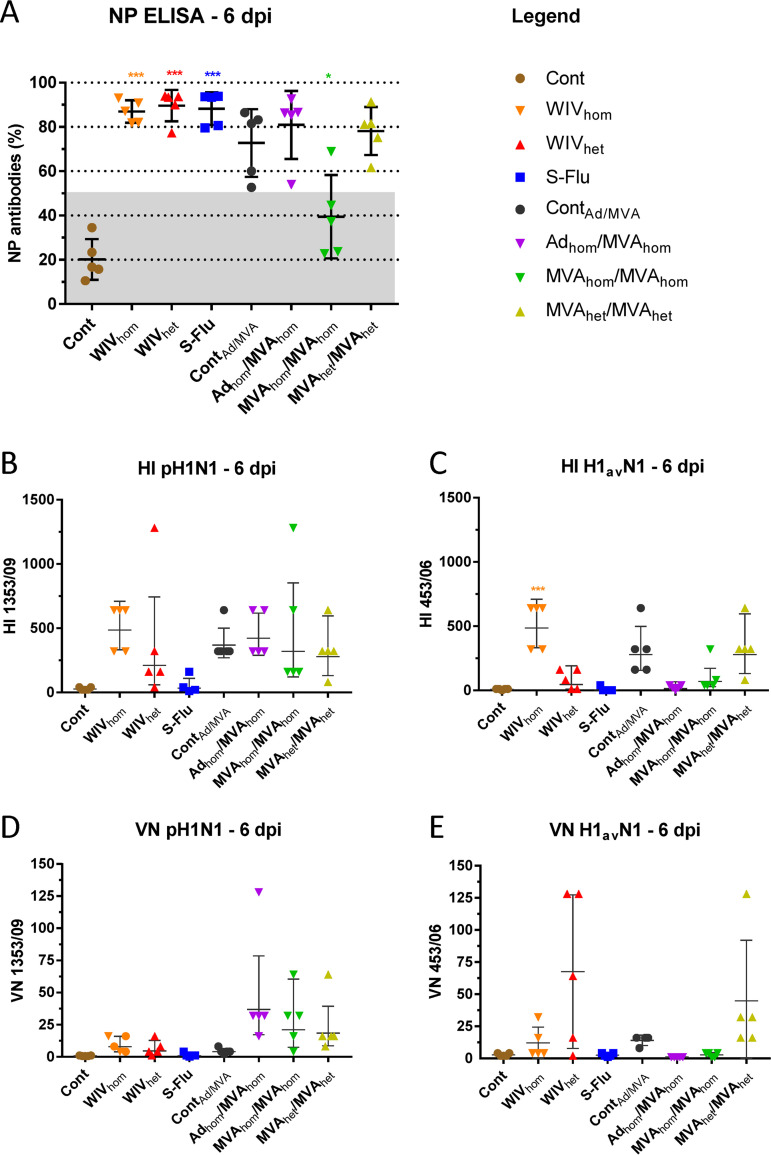
Humoral immune response after challenge infection with pH1N1. Serum samples from 6 days postinfection (6 dpi) were assessed by influenza A virus nucleoprotein (NP) competition ELISA, Hemagglutination inhibition (HI) and virus neutralization (VN) assays. (A) Antibody response to NP is expressed as the inverse of the percentage of competition: 1 − (OD_sample_/OD_negative_) × 100%. Results over 50% are considered positive. Individual HI and VN titers against (B and D) the homologous antigen, pH1N1 (A/swine/England/1353/2009), and (C and E) the heterologous antigen, H1_av_N1 (A/swine/England/453/2006), are shown relative to their corresponding 0-dpv sample. *, *P* < 0.05, and ***, *P* < 0.0001, significant difference from the control group, by the color corresponding to the group, as indicated under “Legend.”

Six days after pH1N1 challenge, anti-NP antibodies were detected in all groups, including the vector control group (Cont_Ad/MVA_). The mean titer increased in the MVA_hom_-immunized group but remained below the threshold of the assay for 4 of the 5 pigs. Only the vehicle control (Cont) group did not have a raised anti-NP response within 6 days postinfection (Fig. 5A). All immunized pigs, except the vehicle control (Cont) and S-FLU groups, showed an increased HI titer to the HA from the pH1N1 challenge strain after infection (Fig. 5B). Evaluation of HI titers to the heterologous H1_av_N1 antigen at 6 dpi (Fig. 5C) showed a low response in the groups receiving the cognate WIV_het_ and MVA_het_ vaccines. Cross-reactive antibodies were elicited in WIV_hom_-vaccinated group (geometric mean titer of 490), as reported previously (9). HI titers were not detected in the S-FLU H3N2 vaccine group, as expected. Interestingly, pigs receiving the control virus-vectored vaccine, Cont_Ad/MVA_, produced cross-reactive antibodies to both HA antigens post-virus inoculation, possibly reflecting generalized priming of the immune system by the vaccine vectors. Six days after pH1N1 challenge, increased neutralizing antibody titers were detected in the Ad_hom_/MVA_hom_ and MVA_hom_ groups, and low levels of neutralizing activity were detected in the heterologous MVA_het_-immunized group (Fig. 5D). An increase in the neutralizing titer to the heterologous H1_av_N1 virus strain was detected in some animals that received the cognate WIV_het_ immunization, as well as animals receiving MVA_het,_ but the antibody did not neutralize the pH1N1 challenge strain (Fig. 5E). Taken together, these results indicate that the heterologous HA antigen from the H1_av_N1 strain was less immunogenic than the homologous pH1N1 HA antigen used in this study, irrespective of whether it was incorporated in an inactivated or virus-vectored vaccine. Additionally, as reported previously (9), a close antigenic match between the vaccine antigen and challenge strain was needed in order to reduce nasal shedding of virus following infection.

### T cell response.

**FIG 6:**
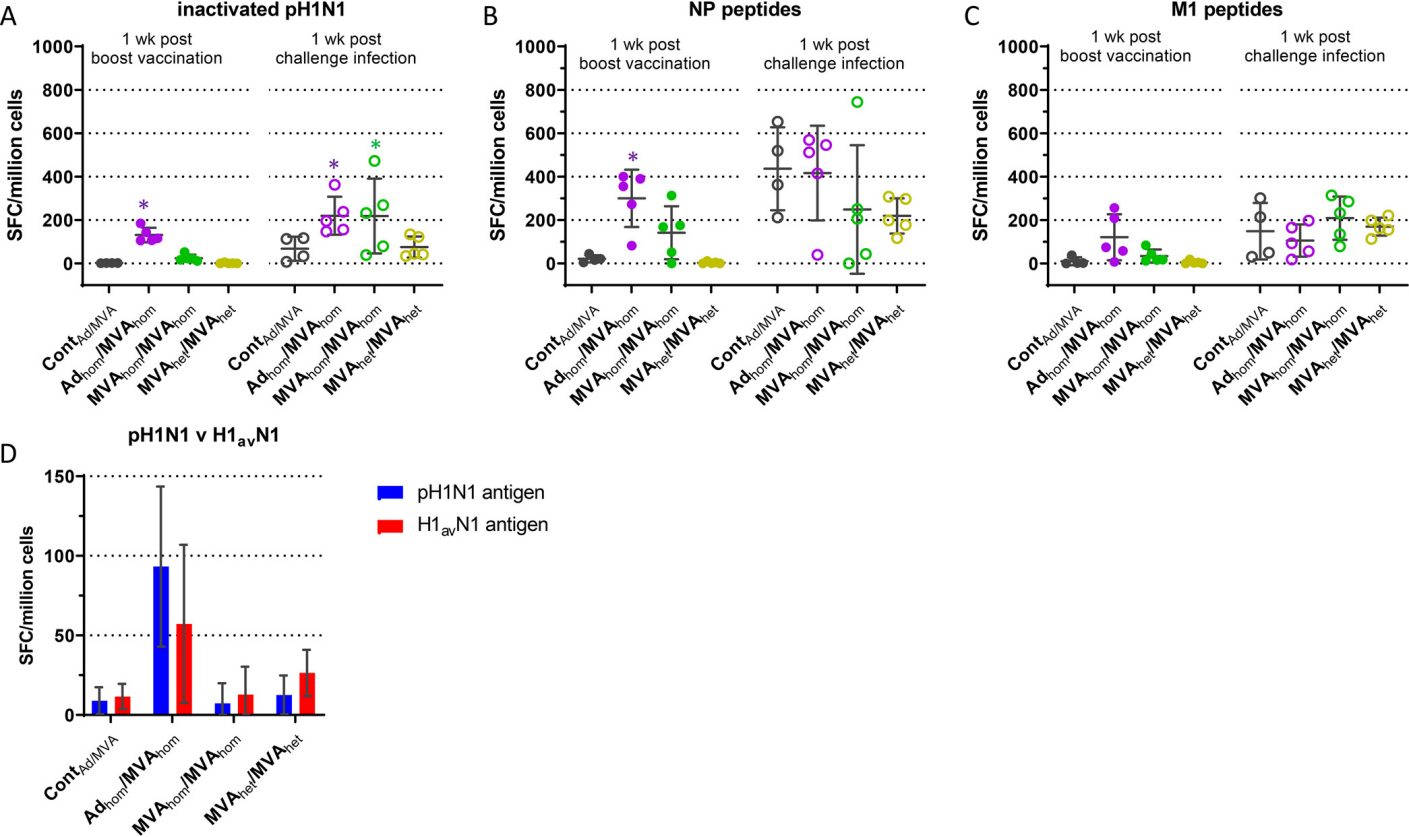
Cellular immune response. Shown is the mean number (± SD) of IFN-γ producing PBMCs (spot-forming cells [SFC]/million cells) induced by (A) inactivated pH1N1 A/swine/England/1353/2009 antigen, (B) NP, or (C) M1 18-mer peptides for the virus-vectored vaccine groups. Responses were evaluated 1 week after boost vaccination (28 dpv [solid circles]) and after viral challenge (6 dpi [open circles]). (D) Comparison of SFC induced by either inactivated homologous antigen (pH1N1 [blue bars]) or heterologous antigen (H1_av_N1 [red bars]) 2 weeks after boost. *, *P* < 0.05, significant difference from the control group.

The cellular immune response, monitored by gamma interferon (IFN-γ) enzyme-linked immunosorbent spot (ELISpot) assay in the virus-vectored vaccine groups, showed that at 1 week postboost (28 dpv), the Ad_hom_/MVA_hom_ immunization elicited the strongest response when peripheral blood mononuclear cells (PBMCs) were stimulated by either the homologous inactivated pH1N1 antigen or the conserved overlapping NP or M1 peptides (means of 131, 300, and 121 spot-forming cells [SFCs], respectively) (Fig. 6A to C). In all four vaccine groups, the number of IFN-γ-producing cells had increased 1 week after the pH1N1 challenge infection (6 dpi) following inactivated-pH1N1 or NP peptide stimulation (Fig. 6A and B). The response to M1 peptides was weaker in all groups (Fig. 6C). Interestingly, MVA_het_ immunization did not induce IFN-γ-producing cells that were stimulated by NP or M1 peptides 1 week after boost, although MVA_het_ contained the same homologous NP and M1 genes as MVA_hom_. Two weeks postboost (35 dpv), reactivity to the inactivated heterologous H1_av_N1 antigen was tested (Fig. 6D). Although responses were low, MVA_het_ animals showed double the response to the cognate H1_av_N1 antigen compared to pH1N1 (26 versus 12 SFCs, respectively), while the MVA_hom_ animals did not show any response different from the control-vaccinated animals (Cont_Ad/MVA_). The Ad_hom_/MVA_hom_-immunized animals had a higher response to the cognate pH1N1 antigen than the heterologous H1_av_N1 antigen (mean of 93 versus 57 SFCs), as expected. Overall, the Ad_hom_/MVA_hom_ combination stimulated the highest cellular immune response, which was most clearly detected using NP peptide stimulation. The other groups displayed lower cellular responses that were more variable, a common finding with commercial outbred animals.

## DISCUSSION

We have carried out a study in pigs to investigate the potential for influenza vaccines to modulate virus shedding following infection, induce protective immune responses, and interrupt transmission to naive animals. We have used a well-established pH1N1 pig infection model, which has proven utility for testing new vaccine platforms with both veterinary and human clinical applications (reviewed in references 4, 8, and 19). In our study, the vaccines homologous to the challenge strain, WIV_hom_, or the virus-vectored combination Ad_hom_/MVA_hom_ reduced virus shedding and delayed, but did not completely prevent, transmission to naive animals when housed in direct contact. These findings suggest that even if infection levels are significantly reduced by an antigenically matched vaccine, thereby slowing both the dissemination of the virus through a herd and the generation time for naive contact pigs, transmission to naive animals may not be entirely interrupted. These findings are in agreement with those from previous reports indicating that vaccines able to significantly limit nasal shedding, irrespective of the vaccine platform used, may delay viral transmission to naive pigs in direct contact and may also decrease the likelihood of indirect transmission (20–22), presumably by lowering viral load in the environment.

Influenza A virus transmission in pigs under field conditions remains poorly understood. To gain further insight, we have recently investigated the kinetics and dynamics of virus transmission in unvaccinated pigs infected by contact exposure (23) and demonstrated that virus transmission occurred on 60% of occasions when infectious virus was detected in nasal swabs. Although infrequent, virus transmission was also identified on four occasions when levels of nasal shedding by donor pigs were lower than 100 PFU/ml, and in one other case, shedding was below the virus titration limit. The latent period for contact pigs was longer when they had been exposed to a smaller amount of shed virus, similar to the results reported here. Conversely, the probability of transmission was found to increase following exposure to a larger amount of shed virus. In addition, following contact transmission, the duration of virus shedding from an infected pig was found to be slightly longer (mean of 4.5 days) than the infectious period (3.9 days) monitored by onward transmission of virus to naive pigs placed in direct contact. Therefore, in a field situation, although the infectious dose for influenza A virus may be low, the infectious period may be slightly shorter than the duration of viral shedding.

Vectored vaccines have the advantage of being able to potentiate the immune response to suboptimal antigens and elicit both humoral and cellular immunity. In our study, WIV_hom_ and the vectored vaccine combination Ad_hom_/MVA_hom_ were the only vaccines that induced robust antibody responses measured by HI and VN. Furthermore, in comparison to these WIV_hom_ and Ad_hom_/MVA_hom_ groups, animals immunized with MVA_hom_ for both prime and boost had significantly lower antibody titers to the pH1N1 strain, and following pH1N1 challenge, the MVA_hom_ group, like the WIV_het_ and MVA_het_ groups, also shed more virus. With respect to the Ad/MVA-vectored vaccines, a prime-boost regimen using vaccines with different vector backbones has been found to be the most efficacious in human clinical trials (17). In our study, H3N2 S-FLU did not reduce viral shedding following pH1N1 challenge virus, despite the ability of this vaccine to induce local cross-reactive T cell response in the lung, as we have shown previously (13). In contrast we have recently shown that a homologous H1N1 S-FLU vaccine administered intramuscularly to pigs induces strong HI and VN antibody response, associated with considerable suppression of shedding after pH1N1 challenge (E. Tchilian, personal communication). Taken together, our data support the notion that a robust humoral, but not T cell, response predicts reduced viral shedding, regardless of the vaccine used to prime the immune response. Furthermore, our findings agree with the view that HI antibody titers do not predict prevention of transmission, although they correlate with reduction of shedding and mitigation of disease (4).

Our studies were extremely stringent because the naive pigs were housed in direct contact with challenged pigs for several days. We were not able to determine whether some contact pigs acquired infection from the immunized and directly challenged pigs and others from the infected contacts. The delay in acquisition of infection by the majority of naive contact pigs in the Ad_hom_/MVA_hom_ group suggests that not all pigs were infected by the challenged pen-mates. It will also be important to determine whether immunized pigs are susceptible to infection as a result of contact with infected pigs, although our preliminary data suggest that this will be an infrequent event. Indeed, another study has demonstrated a significant reduction in the level of virus transmission and the influenza A virus reproduction rate (*R*_0_) within vaccinated relative to unvaccinated animals (24). Field studies also indicate that virus dissemination in a herd can be effectively reduced by use of an antigenically matched vaccine (20–22), but antigenic drift can compromise such control strategies (18, 25).

In summary, it is clear that an influenza vaccine must significantly decrease, if not eliminate, nasal shedding of infectious virus in order to prevent onward transmission from infected pigs, in addition to mitigating clinical disease and/or lung pathology. Often immunization limits disease but does not prevent shedding (6). Consequently, animals remain infectious so that the transmission cycle is not broken and even “immune” pigs, which come into contact with other susceptible animals or humans, may potentially transmit live virus to them. Whether homologous mucosal immunization is more effective in preventing transmission remains to be confirmed in our more stringent model. However, if both the infected and contact pigs are immunized, it is likely that transmission of antigenically matched virus strains will be greatly reduced and clinical disease attenuated.

## MATERIALS AND METHODS

### Ethics statement.

*In vivo* studies were conducted in accordance with U.K. Home Office regulations under the Animals (Scientific Procedures) Act 1986 (ASPA), with protocols approved by the Animal Welfare and Ethical Review Body (AWERB) of the Animal and Plant Health Agency, and the ARRIVE Guidelines were adopted (26).

### Animals.

A total of 80 6-week-old Landrace cross female pigs were obtained from a commercial high-health-status herd in two batches of 40 each. Pigs were screened for absence of influenza A virus RNA by matrix gene RRT-qPCR (27) and absence of influenza A antibodies by hemagglutination inhibition (28) using four swine influenza A virus antigens representative of endemic strains known to be circulating in U.K. pigs, including one pH1N1 antigen. Throughout the study, all pigs received weaner/grower feed and had access to water *ad libitum*. Study pigs were observed daily for signs of illness and/or welfare impairment. For 1 week postvaccination and postchallenge or after cohousing, animals were scored using a clinical scoring system to monitor clinical signs, including demeanor, appetite, and respiratory signs such as coughing and sneezing, as well as rectal temperature (29).

### Viruses and vaccines.

Virus strains used to generate the monovalent, whole inactivated virus (WIV) vaccines were a pandemic swine H1N1 isolate, A/swine/England/1353/2009 (pH1N1) from clade 1A.3.3.2 (30) and a Eurasian avian-like swine H1N1 isolate, A/swine/England/453/2006 (H1_av_N1) from clade 1C.2 (31). Vaccine antigen was prepared from virus propagated in embryonated eggs and inactivated using β-propiolactone (BPL) at (1:2,000) for 2 h as previously described (32). The WIV antigen payload was assessed by the hemagglutination (HA) test, and each dose was formulated in 1 ml with 1,024 HA units (HAU) per ml in an oil-in-water adjuvant, TS6 (https://patents.google.com/patent/WO2005009462A3/en). Vaccines were administered into the trapezius muscle (i.m.), 25 to 30 mm posterior to the ear, using a 1-in., 19-gauge needle.

Virus-vectored vaccines were constructed by the Viral Vector Core Facility of the Jenner Institute, University of Oxford. The modified vaccinia virus Ankara (MVA) vaccines incorporated the complete NP and M1 from A/swine/England/1353/2009 joined by a 7-amino-acid linker sequence and expressed from the vaccinia virus F11 promoter inserted at the F11 locus of MVA (33). The HA coding sequence was derived from the same A/swine/England/1353/2009 or A/swine/England/453/2006 strains used to generate the WIV vaccines and was expressed from the p7.5 promoter at the B8 locus of MVA. Recombinant MVA-vectored vaccines were produced in chicken embryo fibroblast (CEF) cells. All inserts were confirmed by sequencing and were tested for expression and secretion of the proteins by intracellular staining and Western blotting and by ELISA (data not shown). For production of the ChAdOx1-vectored vaccines, expression cassettes were transferred into an adenovirus shuttle plasmid using Gateway technology (Life Technologies); the resulting constructs were linearized by enzyme digestion and transfected into replication-deficient ChAdOx1 as described previously (34, 35). As a control, MVA and ChAdOx1 vaccines incorporating irrelevant antigens for this study, namely, the Zaire Ebola virus surface glycoprotein, were used (36). Each MVA vaccine was administered at a dose of 1.5 × 10^8^ PFU/ml and the ChAdOx1 vaccines at a dose of 5 × 10^8^ IU/ml i.m. in 1 ml, as were the WIV vaccines. The H3N2 S-FLU vaccine is a broadly protective cell-mediated vaccine candidate. This vaccine was constructed with [eGFP*/N2(×217)].H3/Switzerland/9725293/2013 (encoding N2 from A/Victoria/361/2011 from the vaccine strain ×217 and coated with the 3C.3a H3 HA from A/Switzerland/9725293/2013) at 1.52 × 10^8^ 50% tissue culture infectious doses (TCID_50_)/ml (95% confidence interval [CI], 1.13 to 2.05 × 10^8^/ml). The internal protein gene segments were from influenza A/Puerto Rico/8/1934 (H1N1) (13). The animals received ∼1.5 × 10^8^ TCID_50_ in 1 ml in identical prime and boost immunizations with a 21-day interval. The S-FLU vaccine was administered to each animal by aerosol using a vibrating mesh nebulizer (SOLO; Aerogen, Ltd.) attached to a custom-made mask held over the nose and mouth following anesthesia, as described previously (13).

### Experimental design.

Vaccination challenge experiments were conducted with eight groups (G1 to G8) of animals on two separate occasions (Table 1). The same study structure was used (Fig. 1) with pigs housed together during acclimatization for 7 days, the vaccination phase of prime then boost with a 3-week interval and for a further 7 weeks after the boost. In study 1, the following groups were immunized: G1, mock vaccine allantoic fluid vehicle control (Cont) intramuscularly (i.m.); G2, homologous WIV pH1N1 vaccine formulated with TS6 adjuvant i.m. (WIV_hom_); G3, heterologous WIV H1_av_N1 vaccine (WIV_het_) formulated with TS6 adjuvant i.m.; and G4, influenza pseudotype H3N2 administered by aerosol under anesthesia (S-FLU). In study 2, the following groups were immunized: G5, ChAdOx1 prime- and MVA boost-vectored vaccines incorporating the Zaire Ebola virus surface glycoprotein as an irrelevant antigen (Cont_Ad/MVA_) i.m.; G6, ChAdOx1 prime- and MVA boost-vectored vaccines incorporating homologous HA and NP+M1 (Ad_hom_/MVA_hom_); G7, MVA virus-vectored vaccine for the prime and boost incorporating the homologous HA and NP+M1 (MVA_hom_); G8, MVA virus-vectored vaccine for the prime and boost incorporating the heterologous H1_av_-homologous NP+M1 vaccine (MVA_het_). Pigs were housed separately in their vaccine groups for i.n. challenge with 1 × 10^7^ TCID_50_ A/swine/England/1353/2009 (pH1N1) virus in 4 ml using MAD300 (Teleflex) delivery of an atomized spray of droplets with diameters ranging from 30 to 100 µm. Two days later, 5 naive “contact” pigs were housed with each vaccinated/challenged group. Directly inoculated pigs were euthanized at 6 or 7 dpi and naive contact pigs at 12 dpc with an overdose of intravenous pentobarbital sodium.

### Sample collection.

Blood samples (clotted and heparin anticoagulated) were taken prior to vaccination, at 1, 3, 7, 14, 21, 28, 35, 42, 56, and 63 dpv, and at 1, 3, and 6 dpi. Serum was collected and stored at −20°C. PBMCs were isolated and cryopreserved for later determination of the humoral and cellular immune responses triggered by vaccination and/or infection. Nasal swabs (two per nostril) were obtained before vaccination and challenge as well as daily after challenge until the end of the study to determine individual influenza A viral RNA shedding profiles by RRT-qPCR. Swabs were stored dry at −80°C until processing.

### RRT-qPCR.

The nasal swabs from each nasal sample were placed together into 2 ml of Leibovitz L-15 medium (Thermo Fisher Scientific), containing 1% fetal bovine serum (FBS) and 1% penicillin-streptomycin (5,000 U/ml; Gibco). The tubes containing the swabs were agitated, and the supernatant was aliquoted. Total RNA was extracted from these suspensions using the RNeasy minikit (Qiagen, Crawley, United Kingdom) according to the manufacturer's instructions. Viral RNA was detected by RRT-qPCR directed against the influenza A virus M gene (27). RNA quantity is expressed as relative equivalent units (REU) of RNA using a standard 10-fold dilution series of RNA purified from the same batch of virus, of known TCID_50_ titer, used for challenge. Although these units measure the amount of viral RNA present and not infectivity, it may be inferred from the linear relationship with the dilution series that they are proportional to the amount of infectious virus present as described previously (9).

### ELISA.

Antibody titers were measured using a competitive multispecies ELISA to detect the nucleoprotein of influenza A virus (ID Screen IDvet), as well as hemagglutination inhibition (HI) (28) and virus neutralization (VN) (37) assays using homologous and heterologous antigens or viruses.

### Porcine IFN-γ ELISpot assay.

PBMCs were isolated as previously described (38), and IFN-γ-producing PBMCs were assessed in the virus-vectored vaccine groups with an ELISpot assay using High Protein Binding Immobilon-P membrane plates (MAIPS4510; Millipore) and the MabTech Porcine IFN-γ ELISpot kit (3130-2A; MabTech). PBMCs were stimulated with M1 and NP peptides (16, 17) and β-propiolactone-inactivated virus as previously described (9). The number of spot-forming cells (SFCs) was counted with an automated ELISpot reader (AID) and corrected for background (tissue culture medium stimulated).

### Statistical analysis.

Statistical analyses, including calculation of arithmetic means, geometric means, associated standard deviation (SD) or standard error of the mean (SEM), analysis of variance (ANOVA), and associated *post hoc* Tukey’s tests were performed using GraphPad Prism 7 (IBM). Titers and REU values were logarithmically transformed. Geometric mean ratios (GMRs) of titers were calculated from the samples for each pig relative to the baseline 0-dpv sample. Values were compared between groups using two-way ANOVA (repeated measurements), and statistically significance differences were identified using Tukey’s multiple-comparison test. For *k* = 8 groups of *n* = 5 individuals, with power β = 0.80 and type I risk α = 0.05, an effect size of 0.67 could be detected using ANOVA. Differences were considered significant where *P* was <0.05. We defined the epidemiological parameters as previously described (23). Briefly, the latent period is the interval between inoculation or contact and first detection of virus, while the duration of shedding is the interval between first and last sample times when virus is detected. Since the times to first and last virus detection are interval censored, the observed latent period and duration of shedding are an upper limit and lower limit, respectively. We also defined *V*_max_ as the maximal viral titer shed, *T*_max_ as the time to reach *V*_max_, and the generation time, *T_g_*, as the time interval between the onset of virus shedding in a primary case and in its secondary case. The area under the curve (AUC) was evaluated to provide a measure of the total amount of virus shed by an animal. The mean AUC values for each group were collectively compared with the AUC values for all other groups. We assumed that the probability of transmission from a vaccinated pig to a contact pig at time *t*, also called infectiousness, is proportional (with a constant *k*_1_) to viral shedding at *t*, *V*(*t*) (39, 40), as well as homogeneous mixing independent of infection time course to reflect random contacts between the vaccinated and contact pigs (with a contact rate of *k*_2_). Hence, the probability of observing a transmission event at time *t* is given by $P\left(E=1\mathrm{|t}\right)=k_{1}k_{2}V(t)$. The total amount of virus shed by a pig (or exposure) can be computed as the area under the curve:

$$\text{AUC}=\overset{+\infty }{\underset{0}{\int }}V\left(x\right)dx$$

Generation time can then be computed as the expectation of P(E=1|t). We therefore integrated P(E=1|t)t with respect to *t* and normalized by the total rate of transmissions over time, which is *k*_1_*k*_2_ AUC. The expression can be simplified by dropping the constants *k*_1_ and *k*_2_, leading to

$$T_{g}=\overset{+\infty }{\underset{0}{\int }}\frac{t\times V(t)}{\text{AUC}}dt$$

All integrals were computed using the trapezoidal rule as implemented in the caTools package (caTools_1.8.tar.gz; https://cran.r-project.org/web/packages/caTools/caTools.pdf). We presented the estimates as mean ± standard error (SE) for quantitative variables. The effect of vaccine group was tested on the different epidemiological parameters (i.e., latent period, duration of shedding, AUC, and generation time) using first a one-way ANOVA. If the *P* value was <0.05, we then performed a pairwise permutation test as implemented in the rcompanion package (Table 2). Figure 3 was generated using the ggplot2 package (41).
